# VISTA Re-programs Macrophage Biology Through the Combined Regulation of Tolerance and Anti-inflammatory Pathways

**DOI:** 10.3389/fimmu.2020.580187

**Published:** 2020-10-15

**Authors:** Mohamed A. ElTanbouly, Evelien Schaafsma, Nicole C. Smits, Parth Shah, Chao Cheng, Christopher Burns, Bruce R. Blazar, Randolph J. Noelle, Rodwell Mabaera

**Affiliations:** ^1^Department of Microbiology and Immunology, Norris Cotton Cancer Center, Geisel School of Medicine at Dartmouth, Lebanon, NH, United States; ^2^Department of Biomedical Data Science, Geisel School of Medicine at Dartmouth, Hanover, NH, United States; ^3^Department of Medicine, Dartmouth Hitchcock Medical Center, Lebanon, NH, United States; ^4^Department of Medicine, Baylor College of Medicine, Houston, TX, United States; ^5^Department of Laboratory Medicine and Pathology, University of Minnesota, Minneapolis, MN, United States

**Keywords:** VISTA, macrophage, tolerance, immunosuppression, agonist

## Abstract

We present the novel finding that V-domain Ig suppressor of T cell activation (VISTA) negatively regulates innate inflammation through the transcriptional and epigenetic re-programming of macrophages. Representative of VISTA re-programming is the ability of VISTA agonistic antibodies to augment LPS tolerance and reduce septic shock lethality in mice. This anti-inflammatory effect of anti-VISTA was mimicked *in vitro* demonstrating that anti-VISTA treatment caused a significant reduction in LPS-induced IL-12p40, IL-6, CXCL2, and TNF; all hallmark pro-inflammatory mediators of endotoxin shock. Even under conditions that typically “break” LPS tolerance, VISTA agonists sustained a macrophage anti-inflammatory profile. Analysis of the proteomic and transcriptional changes imposed by anti-VISTA show that macrophage re-programming was mediated by a composite profile of mediators involved in both macrophage tolerance induction (IRG1, miR221, A20, IL-10) as well as transcription factors central to driving an anti-inflammatory profile (e.g., IRF5, IRF8, NFKB1). These findings underscore a novel and new activity of VISTA as a negative checkpoint regulator that induces both tolerance and anti-inflammatory programs in macrophages and controls the magnitude of innate inflammation *in vivo*.

## Introduction

Macrophage plasticity plays an important role in controlling both the amplitude and quality of the inflammatory response in a wide variety of physiological and pathological conditions, as well as the resolution of inflammation and tissue repair. To achieve this, macrophages undergo extensive transcriptional and epigenetic reprogramming in response to various environmental cues. These cues allow macrophages to rapidly respond to danger signals by inducing pro-inflammatory mediators on one extreme or to exist in a regulatory state for the purpose of tissue repair and/or maintenance. Two prominent re-programming mechanisms in macrophages that mitigate inflammation are those that mediate the development of tolerance to endotoxin (1) and the alternative differentiation of macrophages to a non-inflammatory phenotype (2).

Lipopolysaccharide (LPS) tolerance is an example of transcriptional and epigenetic reprogramming that prevents macrophage overactivation through development of refractoriness to repeated stimulation resulting in reduced capacity of macrophages to mediate septic shock. LPS tolerance has been extensively studied *in vivo* and *in vitro* with well-documented changes in transcriptional and epigenetic landscapes that abrogates release of the prototypic inflammatory cytokines secreted by activated macrophages, including TNFα, IL-6, IL-1, and IL-12p40. Several mediators including IRAK-M (3), NF-κB1 (p50) (4, 5), mir221/222 (6), IRG1, and A20 (7) have been implicated in mediating or enhancing LPS tolerance.

A second example of macrophage plasticity is historically exemplified by the extremes of M1 (classical) and M2 (alternative) reprogramming of macrophages in response to environmental cues including TLR ligands, cytokines, and other soluble mediators such as corticosteroids and immune complexes [reviewed in Martinez and Gordon (8)]. Compared to the M1 state which is characterized by high production of IL12, TNFα, IL-6, and IL1; various M2 activation states are defined by attenuated production of IL12 and increased production of IL-10 and TGFβ. Key mediators of the M1 program include STAT1, IRF5 (9, 10) and NFKB (8, 11) whereas the M2 programs variably depend on IRF4 (12), NFIL3 (13) and the inhibitory NF-κB homodimers of NF-κB1(p50) and NF-κB2 (p52) (4, 5).

It is clear that the development of the tolerance and anti-inflammatory transcriptional programs have overlapping functional consequences as macrophages polarized toward a regulatory state endow potent protection against LPS-induced lethality (14). In addition, regulatory polarization of macrophages can suppress subsequent pro-inflammatory polarization, and augment tolerance to inflammatory stimuli (2, 14–17). Despite extensive investigations of these two phenomena for many years, little is known about this overlap and how these processes are coordinately regulated *in vivo* to produce a unified macrophage response to a given stimulus. Porta et al. validated that tolerance and alternative macrophage polarization are overlapping transcriptionally regulated processes and showed that NF-κB1 (p50) is central to establishing an “M2-like” state in LPS tolerized macrophages.

Amongst negative checkpoint regulators, VISTA (also known as PD-1H, DD1a, Dies1) is unique in its high levels of constitutive expression on resting myeloid cells, including monocytes and macrophages (18). VISTA is an immunoglobulin superfamily receptor broadly expressed by cells of the hematopoietic compartment (both T cells and myeloid cells) with well-defined roles as a negative immune checkpoint of T cell responses (19, 20). Chen et al. introduced a class of anti-VISTA agonist antibodies and showed in multiple systems, including GVHD and Con A-induced hepatitis, that this class of antibodies suppress T cell mediated immune responses (21–25). Our group further demonstrated VISTA agonistic antibodies also have immunosuppressive activities to ameliorate diseases driven by innate inflammation including antibody-induced arthritis, KBxN arthritis and imiquimod induced psoriasis (20, 26). These findings led to the hypothesis that VISTA may be a negative regulator in the myeloid compartment that tempers the magnitude of myeloid responses to inflammatory stimuli. In this study, we show that VISTA agonists functionally and transcriptionally re-program macrophages by negatively regulating macrophage responses to pro-inflammatory stimuli. Anti-VISTA alone induced mediators involved in both M2 polarization and LPS tolerance including IL-10, miR-221, IRG1, A20, and MerTK and suppressed mediators of M1 polarization (reduced IRF5 and IRF8 expression at both the transcriptional and protein levels). As anticipated, the VISTA-mediated reduction in these transcription factors (TFs) diminished the expression of inflammatory genes including IL-12 family members, IL-6 and TNFα. Furthermore, anti-VISTA upregulated key mediators of LPS tolerance resulting in the enhanced survival of mice from endotoxin shock. In summary, we show that negative checkpoint regulation by VISTA agonists of innate immunity is mediated by the induction of transcriptional reprogramming of both tolerance and anti-inflammatory programs to mitigate innate inflammation *in vivo*.

## Materials and Methods

### Cell Culture

Primary Bone marrow-derived macrophages (BMDMs) were generated by isolation and culture of mouse bone marrow in complete RPMI supplemented with 20 ng/ml recombinant murine M-CSF (Peprotech, 315-02) for up to 7 days. For cell stimulation, 10 ng/ml LPS (Sigma L2630) or 100 ng/ml recombinant mouse IFNγ (Biolegend, 575306) were used. For tolerization experiments, BMDMs (1 × 10^6^ cells/ml per well in a 6 well plate) were stimulated with 10 ng/ml LPS for 15 hours, washed 5 times with 1× PBS, then allowed to rest for 2 h in LPS-free complete medium. BMDMs were then stimulated with 1 μg/ml LPS for 4 h (for total RNA-seq) or 12 h (for Luminex) or as indicated.

For human monocyte and macrophage experiments, Ficoll-Paque (GE Healthcare) was used to isolate PBMCs from healthy volunteers by differential centrifugation. The RPMI 1640 medium (Sigma-Aldrich) was supplemented with 10 mM L-glutamine and 10 mM pyruvate (Life Technologies). Monocytes were obtained by depletion of CD3, CD19, and CD56 positive cells from PBMCs obtained upon Ficoll isolation of a buffy coat. CD3 MicroBeads (130-050- 101), CD19 MicroBeads (130-050-301), and CD56 (130-050-401) were purchased from Miltenyi Biotec and used according to the manufacturer protocol. For RNA-seq analysis of the monocytes, additional CD14 positive cells selection was performed on the CD3-, CD19-, and CD56- population using CD14 MicroBeads (130- 050-201) from Miltenyi Biotec. For human monocyte-derived macrophage differentiation, isolated monocytes were cultured at 2 × 10^6^ cells/ml in 6-well plates (Corning, 3506) in RPMI supplemented with 10% human pooled serum and 20 ng/ml recombinant human M-CSF (Peprotech, 300-25) for 6 days prior to treatment with anti-VISTA for 24 h followed by LPS (1 μg/ml) stimulation. For time-time course RNA-seq analysis, cells were isolated at each time-point, and RNA was extracted as described below.

### Mice

For BMDM generation, hVISTA knock-in mice of 8–10 weeks of age were used (20), unless otherwise noted. Both male and female mice were used in experiments. For tolerance and septic shock experiments, C57Bl/6 mice (Charles River) of 8–10 weeks of age were used. LPS (*Escherichia coli* O55:B5; Sigma L2880) and d-(+)-galactosamine hydrochloride (Sigma G0500) were re-suspended in sterile PBS and filter-sterilized before intraperitoneal injection. Mice were maintained under specific-pathogen–free conditions in the Dartmouth Center for Comparative Medicine and Research. The Animal Care and Use Committee of Dartmouth College approved all animal experiments.

### Antibodies

Anti-VISTA agonist antibodies used in this study were anti-human VISTA clone 803 and anti-mouse VISTA clone 8G8 (20).

### Cytokine Analysis

Simultaneous determination of multiple cytokine concentrations was carried out using the MILLIPLEX MAP Mouse Cytokine/Chemokine Magnetic Bead Panel— Premixed 32 Plex (EMD Millipore, Billerica, MA) on a Bio-Rad Bio-Plex Array Reader. Samples were diluted in cell culture medium to the dynamic range of each kit.

### Proteomic Analysis

Control and anti-VISTA-treated BMDM protein lysate (10 × 10^6^ cells per replicate) we sent for global proteomic quantification (Thermo Fisher Scientific Center for Multiplexed Proteomics at Harvard). In brief, sample were reduced with TCEP, alkylated with iodoacetamide, then quenched with DTT. The proteins were precipitated using methanol/chloroform and sequentially digested with LysC (1:50) and trypsin (1:100) based on protease to protein ratio. Five Hundred milligrams of peptides were labeled for enrichment. Peptides were separated using a gradient of 3 to 25% acetonitrile in 0.125% formic acid over 180 min prior to detection (MS1), sequencing (MS2) in the Ion trap, and quantification (MS3) in the Orbitrap. MS2 spectra were searched using the SEQUEST algorithm against a Uniprot composite database derived from the Mouse proteome containing its reversed complement and known contaminants. Peptide searches were performed using a 20 ppm precursor ion tolerance, 1 Da fragment ion tolerance, Max Internal Cleavage Site: 2, Max differential/Sites: 4, static modifications for TMT tags (+229.163 Da) on Lysine residues and N-terminus peptide, carbamidomethylation (+57.021 Da) on Cysteine residues and a variable modification for oxidation (+15.995 Da) on Methionine residues. For Phosphopeptide searches, another variable modification was considered for phosphorylation (+79.966 Da) on Serine (S), Threonine (T) and Tyrosine (Y) residues. Peptide spectral matches were filtered to a 1% false discovery rate (FDR) using the target-decoy strategy combined with linear discriminant analysis. The proteins were filtered to a <1% FDR. Proteins were quantified only from peptides with a summed SN threshold of >100 and MS2 isolation specificity of 0.5. Quantified proteins were hierarchically clustered using the Euclidean distance, average linkage. Multiple sample test with FDR <0.05 revealed about 1,581 proteins that are significantly changing between two study groups.

### RNA-seq

RNA was extracted using the Kapa Hyperprep with RiboErase kit, according to the manufacturer's instructions. Samples were sequences on the NextSeq500 machine in 75-bp paired-end runs. The quality of the runs was confirmed using the FastQC software (27). Sequencing output files were aligned to GRCh38 and GRCm38 for human and mouse data, respectively. Transcripts were counted by the Spliced Transcripts Alignment to a Reference (STAR) algorithm using the “–quantMode” option (28). The count data matrix was then processed in R and differentially expressed genes (DEGs) were identified using DESeq2 (29). In brief, the data were filtered by removing transcripts that were not detected in all replicates. Differential expression analysis was performed contrasting anti-VISTA-treated samples to the IgG-treated condition. Unless noted otherwise, DEGs were considered to be those with an FDR-adjusted *P* < 0.05. The count data were transformed to log2-transformed transcripts per million (TPM) for downstream analyses and heatmap displays.

Genes differentially expressed throughout the BMDM and human monocyte time-course were selected by three complementary approaches: (1) DESeq2 (29) DEG identification at each time point comparing anti-VISTA to IgG-treatment, (2) EDGE (30, 31) DEG identification comparing the expression dynamics between anti-VISTA to IgG-treatment, (3) ANOVA DEG identification modeled by time and treatment. We selected all genes that were deemed significant by at least two of these methods as differentially expressed throughout the time course.

### scATAC-seq

Nuclei from BMDMs were isolated following the 10X Genomics protocol for scATAC-seq. The CellRanger ATAC v1.1.0 pipeline (32) as used for initial processing. Raw base call (BCL) files were demultiplexed into FASTQ files using “mkfastq.” Reads were aligned to the mouse mm10 reference genome using “count.” Peak count matrices were aggregated into one file using the “aggr” function. Downstream analyses were conducted using the Signac R package (v0.2.4) (33). Only cells considered to be of sufficient quality were retained; cells with at least 3,000 detected fragments, with less that 5% of fragments originating from blacklisted regions, with more than 20% of all fragments mapping to gene peaks, with nucleosome binding patterns present (nucleosome_signal < 10) and with a transcriptional start site (TSS) enrichment score of at least 2 were considered of high quality. The remaining cells were normalized for sequencing depth using frequency-inverse document frequency (TF-IDF) normalization. Singular value decomposition (SVD) was used to reduce the dimensionality of the data. Since the first reduced component was highly correlated with sequencing depth (pearson correlation coefficient = −0.97), only the second to 30th components were retained for further analyses. Unsupervised clustering using Uniform Manifold Approximation and Projection (UMAP) (34) was used for all visual presentations of the data using the “RunUMAP” function on SVD-reduced data and the aforementioned components. Cell clusters were identified using the find “FindClusters” function using resolution 0.3. Cluster marker genes were obtained by the “FindAllMarkers” function using a logistic regression framework to determine differentially expressed genes. Markers with a Bonferroni corrected *p* < 0.001 were considered true marker genes. For global comparisons between treatment groups, the “FindAllMarkers” function was similarly used after using “SetIdent” to specify the treatment identify for each cell. A gene activity matrix was generated to evaluate gene-level differences between treatments. Gene coordinates for the mouse genome were obtained from EnsembleDB with the EnsDb.Mmusculus.v79 R package (v2.99.0) (35). Gene regions were extended to include the 2kb upstream promoter region. Gene activities were assigned based on the number of fragments that mapped to each of the gene regions using the “FeatureMatrix” function. Gene activity scores were log normalized using the “NormalizeData” function. The gene activity scores were utilized for all presented heatmaps.

### GSEA, TF Enrichment and Network Display

Gene Set Enrichment Analysis (GSEA) was performed using the GSEA software provided by the Broad (36, 37) (v4.3.0). Pathway gene sets were downloaded from the C2 and C7 category of the Molecular Signatures Database (MSigDB v7.0) database (36, 37). Only gene sets with at least 10 effective genes (i.e., the number of genes presented in a gene expression dataset) were retained. Transcription factor (TF) target genes were obtained from TRRUST (v.2), a manually curated database of human and mouse transcriptional regulatory networks (38). In addition, TF targets were added manually based on a literature investigation of TFs of interest. The TF network was displayed using Cytoscape (39).

## Results

### Anti-VISTA Enhances LPS Tolerance and Enhances Resistance to Septic Shock

**Figure 1:**
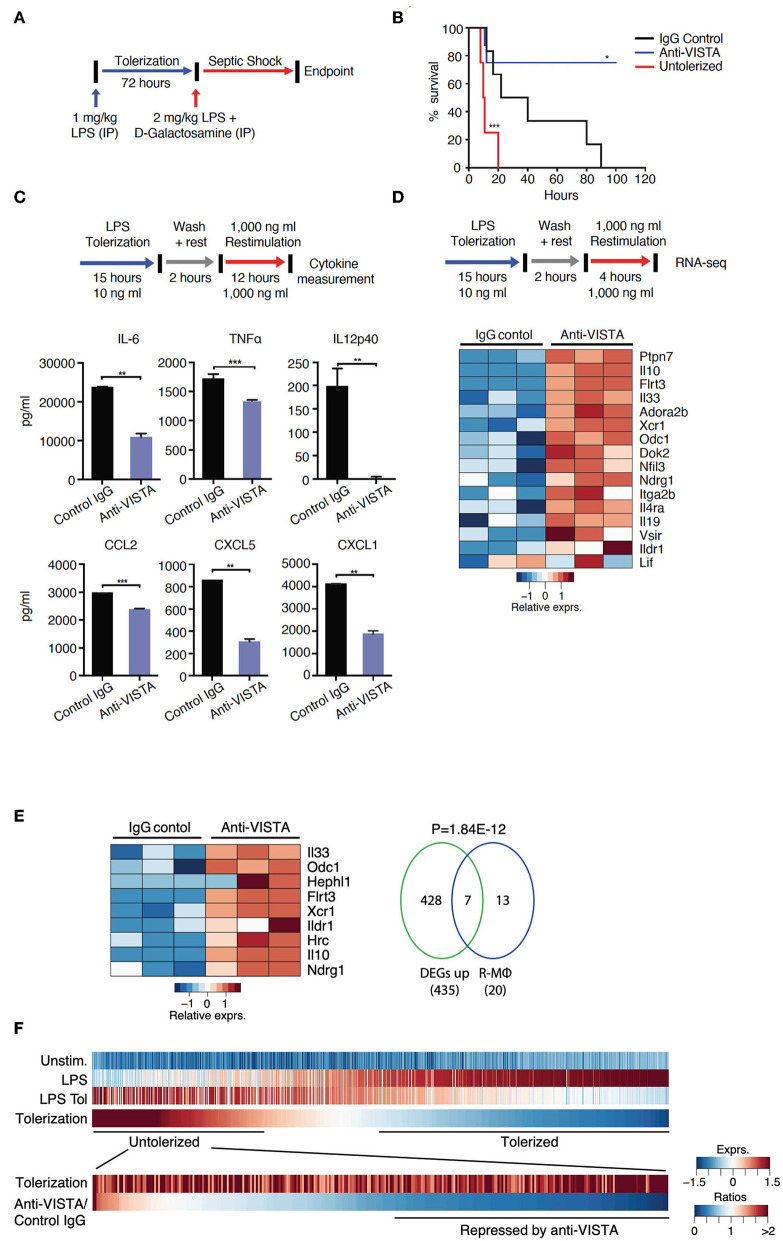
VISTA targeting augments LPS tolerance *in vivo* and *in vitro*. **(A)** Anti-VISTA enhances LPS-induced tolerance in a model of LPS-induced septic shock. Mice were partially tolerized using 1 mg/kg LPS in the presence of anti-VISTA or control IgG for 72 h followed by the induction of septic shock using 2 mg/kg LPS + D-galactosamine and monitored for survival (top). **(B)** Survival of mice following treatment with control Ig, anti-VISTA or non-tolerized (*n* = 8/group in the antibody treatment and *n* = 4 for untolerized mice control). This experiment is a representative of 3 independent repeats with *p*-values calculated by log rank test (bottom). *P*-value of anti-VISTA treatment vs. Isotype IgG control treatment is 0.0194 whereas *p*-value of untolerized compared to antibody treatment is 0.0004 **(C)** Anti-VISTA enhances a tolerogenic profile in LPS-treated BMDMs *in vitro*. Anti-VISTA or control Ig-treated BMDMs (1 × 10^6^ cells/ml per well in a 6 well plate) were tolerized by treatment with 10 ng/ml LPS for 15 h, washed and rested for 2 h, then stimulated by 1 μg/ml LPS for 12 h. Luminex analysis was performed on supernatant. This data is representative of three independent repeats with three biological samples of pooled BMDMs. Each bar indicates the mean value, and each error bar refers to one standard deviation (SD). Student's *t*-tests were performed on anti-VISTA vs. Control IgG samples. **(D)** Differential gene expression in anti-VISTA treated LPS activated BMDMs. BMDMs were treated for 15 h with LPS and control Ig or anti-VISTA, rested for 2 h and restimulated with LPS (top). Heat map of RNA expression (RNA-seq) of selected differentially expressed genes from anti-VISTA vs. control IgG treated BMDMs after 4 h of restimulation with LPS following LPS tolerance (bottom)**. (E)** Anti-VISTA induces a regulatory macrophage transcriptional profile. Comparison between Anti-VISTA treated tolerized BMDM profile vs. Regulatory BMDM previously reported (14). *P*-value calculated by hypergeometric test. These experiments are representative of three independent repeats with three biological samples per repeat. **(F)** *Anti-VISTA expands the breadth of LPS tolerizable genes*. Genes induced by LPS stimulation (“LPS”) compared to unstimulated (“Unstim.”) were identified and classified as non-tolerized (red) or tolerized by LPS pretreatment (blue) (“LPS Tol”) in BMDMs (as described in **(C)**. The extend of tolerization was determined by the ratio of “LPS tol” and “LPS” (“Tolerized”). Genes non-tolerized by LPS were further evaluated for expression upon anti-VISTA treatment (“Anti-VISTA/Control IgG”). Statistical significance of *P ≤ 0.05, **P ≤ 0.01, ***P ≤ 0.001, whereas ****P ≤ 0.0001.

Endotoxin shock is a well-established model wherein a high-dose LPS injection induces a sterile inflammatory shock resulting in macrophage production of TNFα, IL1 and other cytokines (40–44) and subsequent lethality (45, 46). Furthermore, it is well-established that prevention of endotoxin shock can be induced by the prior treatment of the host with low dose LPS. Based on prior studies that showed that anti-VISTA could diminish innate inflammation, initial studies were designed to determine if anti-VISTA could enhance LPS tolerance. Under conditions of partial tolerance induction by LPS (Figure 1A), VISTA agonistic mAb treatment conferred remarkably enhanced protection against LPS-induced lethality (Figure 1B) (47). However, this enhancement required concurrent administration of low-dose LPS since pretreatment with [even multiple doses] anti-VISTA alone did not confer significant protection to high dose LPS (Supplementary Figure 1).

To gain insights into the reprogramming that was conferred by anti-VISTA, a well-established *in vitro* system of LPS tolerance on purified bone-marrow derived macrophages (BMDMs) was used. In these studies, initial stimulation of BMDMs with low-dose LPS induces a tolerogenic form of innate immune re-programming that results in reduced responsiveness to subsequent stimulation with high-dose LPS (47–51). Under these conditions of LPS tolerance, anti-VISTA treatment enhanced tolerance based on significant reductions in IL-12p40, IL-6, CXCL2, and TNFα; all hallmark cytokines for LPS-induced endotoxin shock (Figure 1C) (11, 52–54). These findings show that anti-VISTA synergizes with low dose LPS to expand a program that reduces the production of pro-inflammatory cytokines.

The transcriptional program that results in LPS-induced tolerance in macrophages has been well-established. To rigorously define the components of this program that anti-VISTA modulates to enhance LPS-induced tolerance, the transcriptional profile of BMDMs stimulated *in vitro* by concurrent treatment with LPS with control Ig or with anti-VISTA was analyzed. This analysis revealed that anti-VISTA and LPS induced a regulatory macrophage program (Figure 1D) (20). This regulatory macrophage program was previously reported as a unique set of common transcripts induced in macrophages stimulated by immunomodulatory agonists [e.g., Prostaglandin E2 (PGE2), Aldosterone (Ado)] leading to macrophages that were anti-inflammatory and protected mice from septic shock (14). We report that this set of genes is enhanced by anti-VISTA and LPS when compared to control Ig and LPS (Figure 1E). One hallmark was the upregulation of *Nfil3*; a transcription factor that directly upregulates *Il10* and suppresses *Il12* gene expression, respectively (Figure 1D) (13, 55, 56). This was also concomitant with the upregulation of its target IL-10, as well as SHP-1 (*Ptpn7*), and Flrt3; all effectors of macrophage regulatory activation and anti-inflammatory response (14, 57). Hence, concurrent VISTA engagement alters the trajectory of LPS stimulated macrophages to divert to a less pro-inflammatory profile and contributes to the ability of anti-VISTA to enhance LPS tolerance *in vivo*.

### Anti-VISTA Expands the Breadth of LPS Tolerizable Genes

Numerous studies have identified tolerizable and non-tolerizable genes in systems of LPS tolerance. The previous data presented (Figure 1) show that anti-VISTA can augment the magnitude of tolerance induced by LPS and therefore an analysis was performed to define the registry of tolerizable vs. non-tolerizable genes induced by concurrent LPS and anti-VISTA. One thousand, two hundred twenty-eight genes were identified to be strongly induced by the primary stimulation with LPS of which the expression of 878 genes was reduced (tolerized) and 350 genes were re-induced (untolerized) upon re-stimulation with LPS (Figure 1F). Importantly, half of the identified LPS untolerizable genes were repressed by anti-VISTA treatment, confirming a broadening of the genes suppressed by the concurrent presence of anti-VISTA during the induction of LPS tolerance. TF enrichment analysis of these genes yielded significant enrichments for NFkB1, Rel, and Rela (Supplementary Table 1); all TFs with an established role in macrophage pro-inflammatory reprogramming in response to LPS (58, 59). These findings provide molecular insights into how VISTA agonism imparts a regulatory profile on the macrophages by restraining the expression of effectors of inflammatory “M1” polarization. Multiple analyses highlight a downregulation of NFkB1, REL, and IRF5 at the levels of expression and activity with anti-VISTA causing muted pro-inflammatory polarization as marked by reduced induction of their target genes. This led to a reduction in LPS response pathways and a skewing toward an unstimulated cell state after LPS activation.

### Anti-VISTA Alters the Epigenetic Profile of Tolerized Macrophages

**Figure 2:**
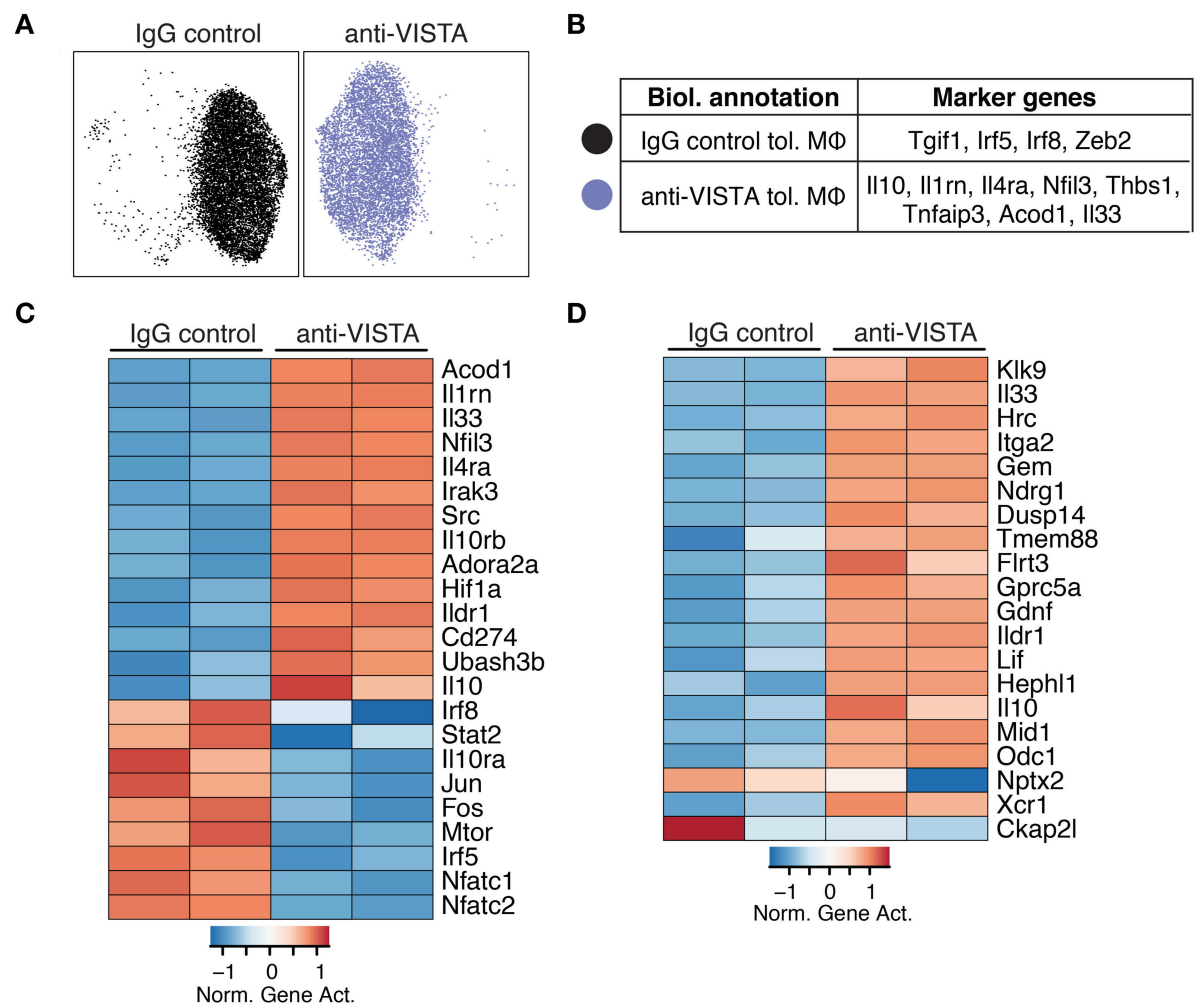
Anti-VISTA alters the epigenetic profile of tolerized BMDM. **(A)** Anti-VISTA alters the epigenetic steady state of tolerized BMDMs. UMAP plot of scATAC-seq on anti-VISTA agonist or IgG isotype control treated LPS-tolerized BMDMs 15 h after treatment with tolerizing LPS dose (10 ng/ml). Data is representative of ~10,000 cells from 2 independent biological samples per group. **(B)** Representative genes that define the clusters identified by scATAC-seq analysis presented in **(A)**. **(C)** Anti-VISTA induces global changes in the epigenetic profile of tolerized BMDMs. Heatmap presenting global differences in gene activity between anti-VISTA vs. IgG control in LPS-tolerized BMDMs. **(D)** Anti-VISTA induced a regulatory macrophage profile. Comparison between Anti-VISTA treated tolerized BMDM profile vs. Regulatory BMDM previously reported (14). This data is representative of two independent repeats with two biological samples per group for each repeat.

Given that LPS tolerance in macrophages is evident at the epigenetic level (47, 48), we examined whether anti-VISTA treatment augmented the epigenetic tolerogenic programming of macrophages in response to LPS tolerization. Analysis of the chromatin accessibility by ATAC-seq revealed a striking difference imposed by anti-VISTA treatment in the context of LPS tolerance (Figure 2A). Unsupervised clustering identified two cell states in LPS tolerized macrophages, where anti-VISTA induced a regulatory macrophage profile highlighted by the enhanced differential accessibility to *Il1rn, Socs3, Il10, Nfil3* and other genes upregulated in anti-inflammatory macrophages (Figure 2B). On the other hand, we also observed reduced accessibility to macrophage polarizing factors such as *Irf5, Irf8*, and *Tgif1* (Figure 2B). Global epigenetic analysis supported these differences as anti-VISTA treatment of LPS-tolerized macrophages profoundly enhanced their tolerogenic phenotype as marked by enhanced gene activity of *Il10, Il1rn, Nfil3* as well as multiple genes upregulated by regulatory macrophages such as *Ildr1* and *Flrt3*, in direct support of the RNA-seq data (Figure 2D). As observed in the RNA-seq analysis, the epigenetic profile of the VISTA activated macrophages overlapped with the transcriptional signature of regulatory macrophages (Figure 2D) (14). These findings suggest that anti-VISTA agonism amplifies macrophage LPS tolerance at the epigenetic level.

### Anti-VISTA Reprograms the Subsequent Inflammatory Response to LPS

**Figure 3:**
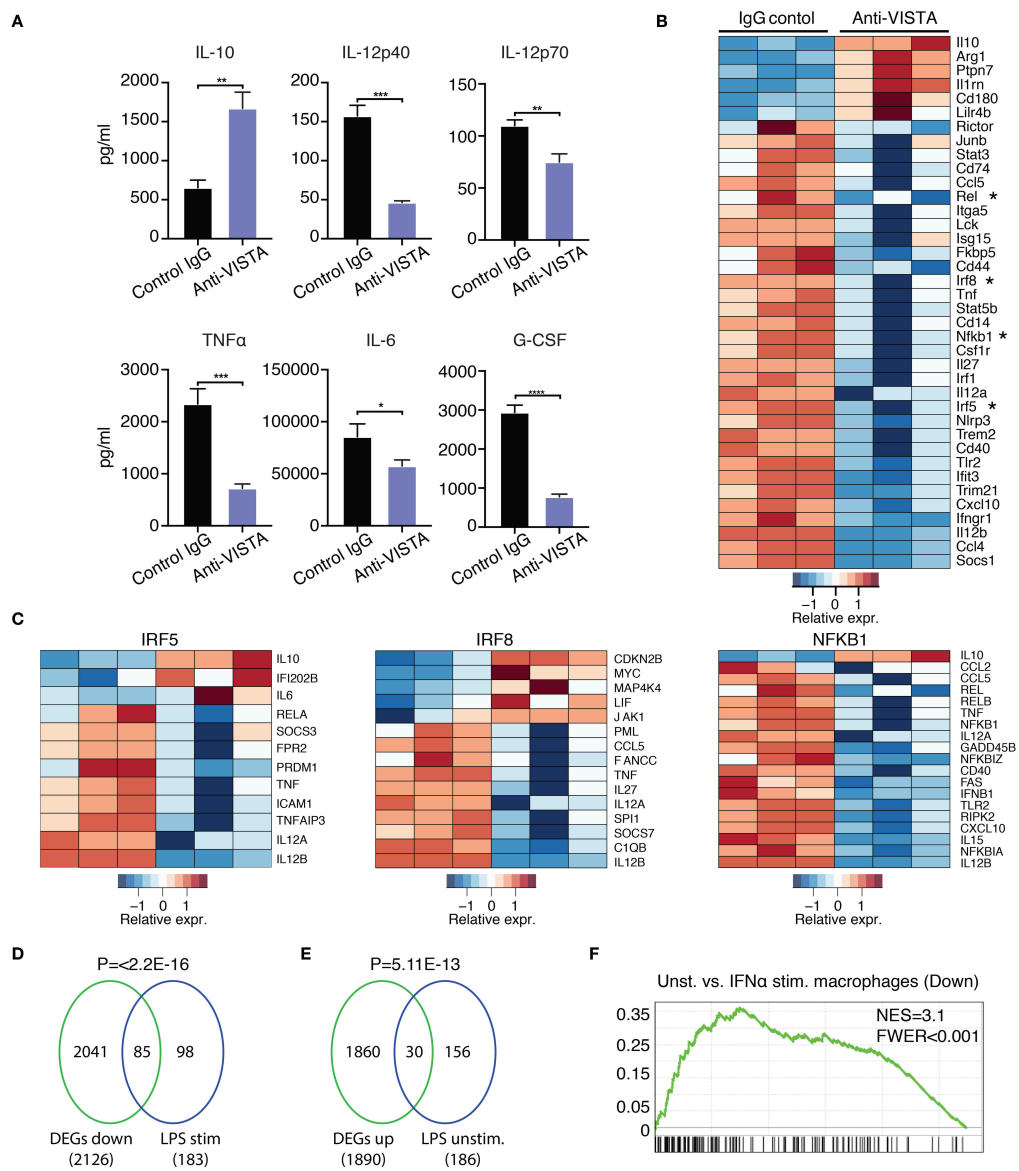
Anti-VISTA induces LPS tolerance based on changes in cytokine production and transcriptional profile. **(A)** Pretreatment with anti-VISTA alters cytokine production by LPS-activated BMDMs. BMDMs were treated with anti-VISTA vs. Control IgG for 24 h then stimulated with 1 μg/ml LPS for 24 h and supernatant analyzed by mouse 32-plex. Bar graphs presents average cytokine levels from 4 biological samples of pooled BMDM. Each bar indicates the mean value, and each error bar refers to one standard deviation (SD). Student's *t*-tests were performed on anti-VISTA vs. Control IgG samples. **(B)** Pretreatment with anti-VISTA alters the transcriptional profile of LPS-activated BMDMs to a tolerized macrophage phenotype. Shown is a heatmap of RNA-seq analysis on anti-VISTA vs. Control IgG pretreated BMDMs after 4 h of acute stimulation with LPS (as in **A**). **(C)** Heat maps of TF target gene expression for IRF5, IRF8, and NFkB1 in BMDMs treated with anti-VISTA vs. Control IgG after 4 h of acute stimulation with LPS. **(D)** Enrichment analysis comparing the downregulated transcriptional profile of Anti-VISTA agonist and LPS stimulated macrophages to the well-defined profile induced upon LPS stimulation (48) and **(E)** enrichment analysis comparing the upregulated gene profile of Anti-VISTA and LPS stimulated macrophages to genes expressed in naïve unstimulated macrophages (48). Anti-VISTA downregulates genes induced by LPS and **(E)** enriches for genes expressed in naïve macrophages. *P*-value was calculated by a sample permutation test (GSEA). **(F)** Gene-set Enrichment analysis (GSEA) output of anti-VISTA and LPS treated macrophages compared to control treatment (IgG + LPS) indicating a significant enrichment of genes upregulated by unstimulated macrophages compared to IFN-a stimulated macrophages in genes upregulated in unstimulated macrophages in the anti-VISTA and LPS treated group. Anti-VISTA imposes a generalized anti-inflammatory profile in pretreated BMDMs. Cytokine measurement experiments are representative of four independent experiments with at least three biological samples per experiment. RNA-seq analysis is representative of two independent experiments with at least three biological sample per experiment. Statistical significance of *P ≤ 0.05, **P ≤ 0.01, ***P ≤ 0.001, ****P ≤ 0.0001.

Prior data presented show that the concurrent treatment of macrophages with LPS and anti-VISTA altered the transcriptional and epigenetic trajectories of their tolerogenic/regulatory profile (Figures 1, 2). Given the striking impact of anti-VISTA on LPS tolerance, we anticipated that prior treatment with anti-VISTA could re-program macrophages to differentially respond to a subsequent cytokine response to LPS. To this end, BMDMs were pretreated with anti-VISTA or control IgG for 24 h then stimulated with LPS for 24 h. As shown, the pretreatment with anti-VISTA agonist caused significant upregulation of the immunomodulatory cytokine IL-10 and suppression of pro-inflammatory cytokines such as IL-12 family (IL12p40, IL12p70), TNFα, IL-6, and G-CSF (Figure 3A). This is distinct from studies shown in Figure 1, in that low-dose LPS was not used to tolerize the macrophages. Analysis of the transcriptional impact of anti-VISTA on the subsequent LPS response showed a clear impact of anti-VISTA on suppressing the expression of cytokines *Il12a, Il12b, Tnf*, *Cxcl10* while upregulating anti-inflammatory mediators including *Il10, Ptpn7*, and *Il1rn*. This profile of changes induced by anti-VISTA is consistent with the development of a tolerized macrophage phenotype (Figure 3B). The gene expression of transcription factors (TF) Irf5, Irf8, Rel, and NFkB1 were significantly reduced (Figure 2B) and the reduction in the activity of these TFs was confirmed by TF enrichment analysis (Figure 3C, Supplementary Figure 2A, Supplementary Table 2). IRF5 plays a critical role in macrophage inflammatory polarization, as it influences macrophage activation toward an inflammatory trajectory by direct upregulation of IL-12 and repression of IL-10 genes (9, 60). IRF8 plays similar roles in pro-inflammatory programming of macrophage polarization (61, 62). More recent work showed that IRF5 interaction with NFkB (Rel-a) plays a substantial role in the induction of inflammatory genes upon LPS stimulation (63). Therefore, downregulation of IRF5, NFkB1, and IRF8 by anti-VISTA treatment followed by LPS stimulation explains the subsequent downregulation of their target genes. The profile of VISTA reprogrammed macrophages with subsequent LPS stimulation was compared to the profile of tolerized vs. untolerized macrophages previously reported by Medzhitov and colleagues (48). In the VISTA agonist group, enrichment analysis showed a marked downregulation of genes induced in macrophages stimulated by LPS (Figure 3D). In addition, VISTA triggering upregulated genes that were enriched in naïve unstimulated macrophages (Figure 3E). VISTA agonism also significantly enriched for genes in LPS tolerized macrophages (Supplementary Figures 2A,B). We also observed a significant overlap in genes downregulated upon IFN stimulation (Figure 3F) and for multiple other inflammatory response pathways (Supplementary Figure 2C), suggesting an overall anti-inflammatory transcriptional profile elicited by anti-VISTA.

**Figure 4:**
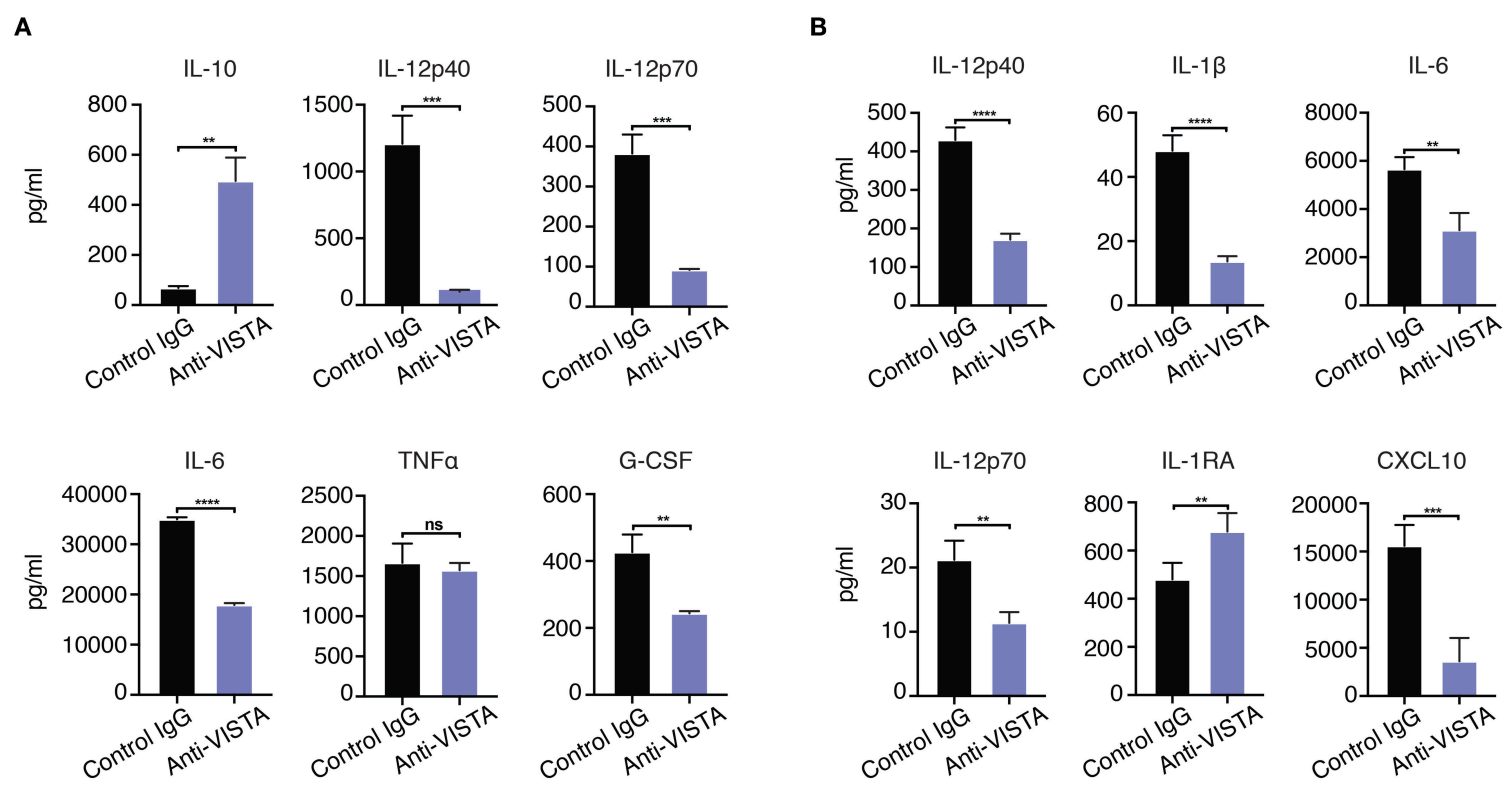
**(A)** Anti-VISTA overrides IFN-γ reversal of LPS tolerance. BMDMs were treated with anti-VISTA vs Control followed by stimulation with LPS (100 ng/ml) and IFN-y (100 U/ml) and cytokines were measured by Luminex. This data is representative of three independent repeats with three biological samples of pooled BMDMs. Each bar indicates the mean value, and each error bar refers to one standard deviation (SD). Student's *t*-tests were performed on anti-VISTA vs Control IgG samples. **(B)** Anti-VISTA induces a tolerogenic cytokine profile in human monocyte-derived macrophages. Isolated monocytes were differentiated to macrophages for 6 days prior and treatment with anti-hVISTA or control Ig (hIgG2) for 24 h followed by LPS (1 μg/ml) stimulation. Supernatant analysis of cytokines from anti-VISTA or control pretreated human macrophages that were stimulated is shown. The data is representative of three independent repeats from 1 healthy donor per repeat. Statistical significance of *P ≤ 0.05, **P ≤ 0.01, ***P ≤ 0.001, ****P ≤ 0.0001.

Given the *in vitro* impact of anti-VISTA on the enhanced breadth of anti-inflammatory related genes, the findings suggest that anti-VISTA could instill a more stable, penetrant and committed anti-inflammatory program. Interferon-gamma (IFN-γ) is a potent macrophage activation factor that augments responses to TLR ligands including LPS (64). One well-established implication of this activity is that IFN-γ can prevent endotoxin tolerance, and restore inflammatory cytokine production in response to LPS in both humans and mice (65–69). Therefore, we tested the impact of IFN-γ on VISTA-induced programming of regulatory macrophages in the presence of LPS stimulation. Strikingly, anti-VISTA pretreatment maintains its suppression of macrophage pro-inflammatory response to LPS in the presence of IFN-γ (Figure 4A). These findings suggest that VISTA triggering can supersede the breach in endotoxin tolerance mediated by IFN-γ and sustain a regulatory program in macrophages even under rigorous conditions of pro-inflammatory polarization. Studies were expanded to address if the tolerogenic/anti-inflammatory re-programming seen in mouse BMDMs by anti-VISTA was also apparent in human macrophages stimulated with anti-human VISTA. Even under conditions of direct acute LPS stimulation, pretreatment with anti-VISTA also induced a reduction in pro-inflammatory cytokines IL-6, TNFα and IL12p40 and an increase in IL-10 which supports our initial contention that anti-VISTA alone can confer a regulatory program on macrophages (Figure 4B).

### Comparative Analysis of Anti-VISTA Alterations in the Proteome and Transcriptome of Human and Mouse Macrophages

**Figure 5:**
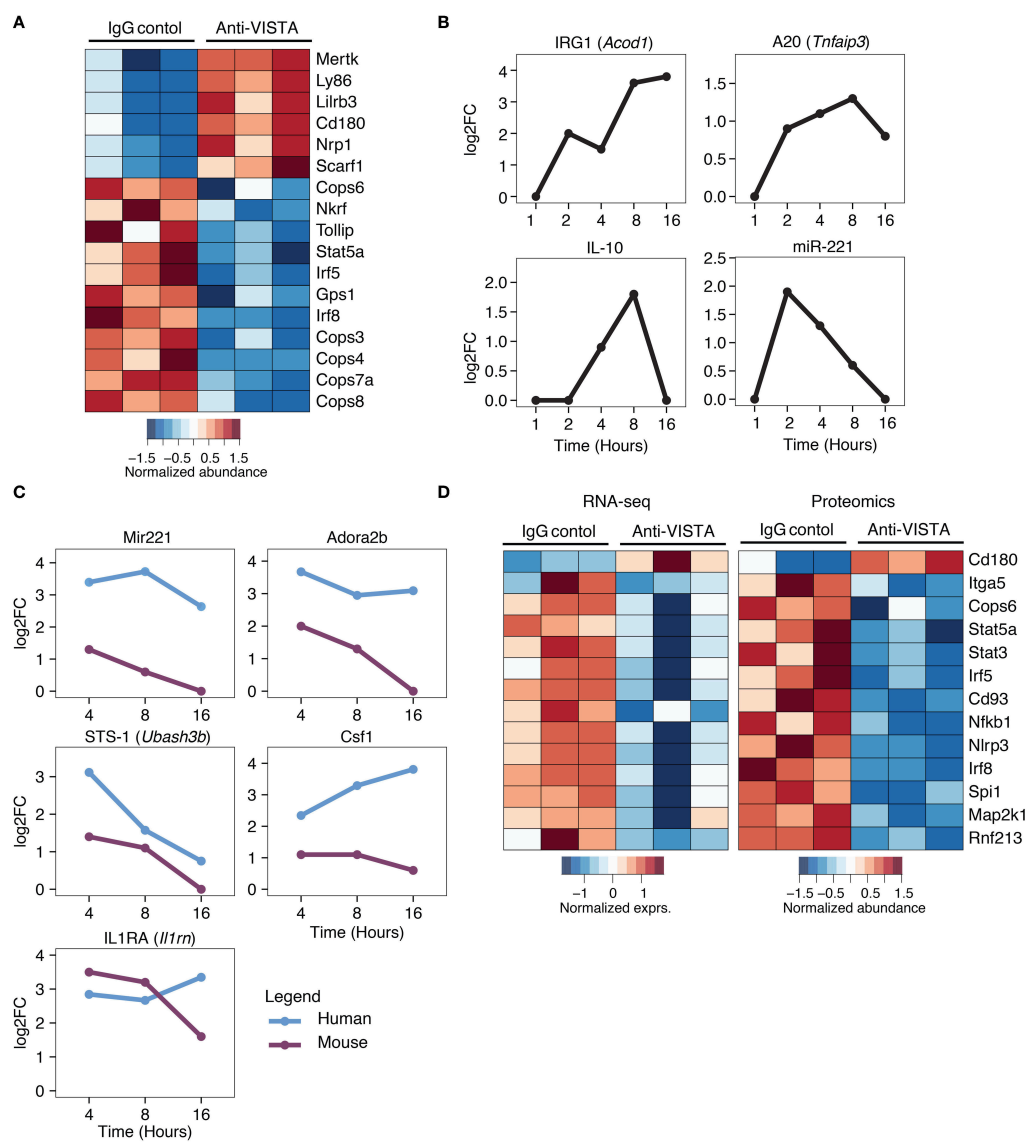
Comparative analysis of anti-VISTA alterations in the proteome and transcriptome of human monocytes and mouse macrophages. **(A)** Anti-VISTA agonist induces a tolerogenic proteomic profile in BMDMs. Heatmap presenting quantified global proteomic changes in BMDMs treated with anti-VISTA agonist (803) for 30 min (details in Methods). Multiple sample test with FDR <0.05 revealed 1,581 proteins that were significantly changing between two study groups. Data is representative of compiled three biological independent repeats of pooled BMDMs (10 × 10^6^ cells per sample). **(B)** Anti-VISTA induced changes in the transcriptional expression of genes involved with LPS-induced macrophage tolerance. Kinetics of mRNA expression of the genes Acod1, Tnfaip3, Il10, and miR-221 from a time-course assessment of anti-VISTA treatment of BMDMs at 1, 2, 4, 8, and 16 h by total RNA-seq. **(C)** Comparative analysis of anti-VISTA alterations in the transcriptional expression of miR-221, Adora2b, Ubash3b, Csf1, and Il1rn in human and mouse macrophages. The log2 fold change (log2FC) of differentially expressed genes comparing Anti-VISTA agonist and Control IgG-treated BMDM and monocyte derived human macrophage were compared. Kinetics of mRNA expression of the genes miR-221, Adora2b, Ubash3b, Csf1, and Il1rn upregulated by both mouse BMDMs and human monocytes after anti-VISTA treatment. **(D)** Anti-VISTA induces similar changes in gene expression when analyzed at both the transcriptional and proteomic levels. Heatmap presenting common genes differentially expressed after anti-VISTA treatment at both the proteomic level from the same dataset in **(A)** and by RNA-seq after LPS stimulation (from data presented in Figure 3).

Given the profound impact of anti-VISTA agonistic antibodies in mitigating myeloid driven inflammatory disease and LPS-induced inflammatory mediators, we sought to investigate the transcriptional and proteomic changes induced by anti-VISTA alone in both mouse and human macrophages. Proteomic analysis on BMDMs after 30 min of anti-VISTA treatment showed significant reduction multiple key pro-inflammatory mediators including NFkB1, IRF5, and IRF8 (Figure 5A). In contrast, the levels of factors involved in macrophage regulatory activity such as MerTK, LILRB3, and NRP1 were all upregulated after anti-VISTA treatment (Figure 5A). More insights into the regulatory program imposed by anti-VISTA treatment was afforded using time-course RNA-seq analysis of BMDMs compared to control treatment. VISTA triggering resulted in a profound induction of several well-established effectors of macrophage tolerance including IRG1 (*Acod1*) and its downstream effector NFkB inhibitor A20 (*Tnfaip3*), miR-221, Il1RA, and IL-10. By 16 h, IRG1 was the top upregulated gene in the VISTA-treated macrophages (Figure 5B). IRG1 is upregulated during endotoxin tolerance and plays an important role in augmenting macrophage tolerance and inhibition of TLR responses, in part by upregulating A20, an inhibitor of NFkB signaling (7, 70). In agreement with this, the chromatin accessibility state of *Acod1, Il1rn, Il10*, and its regulator *Nfil3* was significantly enhanced with anti-VISTA agonist treatment as revealed by ATAC-seq (Supplementary Figure 3).

Time-course analysis of anti-VISTA treated human monocytes and mouse macrophages revealed consistent trends of upregulation of immunomodulatory genes including miR-221, Adora2b, STS-1 (*Ubash3b*), and IL1RA (*Il1rn*) (Figure 5C) (47, 71–75). Time-course pathway analysis VISTA agonist-treated human monocytes revealed a remarkable downregulation of multiple inflammatory response pathways, and this downregulation was also observed in BMDMs (Supplementary Figure 4A,B). TF enrichment analysis of genes that showed significant changes in expression revealed that NFKB1, and RelA targets were significantly enriched among downregulated genes (Supplementary Figure 4C, Supplementary Table 3). This was associated with transcriptional suppression of several key transcription factors (TF) involved in macrophage inflammatory programing including IRF5, IRF8, and NFkB1 (Figure 5D). Given the importance of these factors in driving macrophage inflammatory responses, the data show that VISTA agonists strategically alter the macrophage transcriptome to resist polarization to an inflammatory state (Figure 5D).

## Discussion

The expression of VISTA by myeloid lineage cells is broad and constitutive. Published studies show that the genetic deletion of VISTA results in heightened steady-state myeloid activation and the production of immune mediators (19, 24, 76, 77). Therefore, VISTA is a negative checkpoint regulator whose constitutive function is to keep the myeloid compartment immunologically “quiet.” Data presented in this study show that in addition to this constitutive function, VISTA also plays a role during inflammatory challenges to re-program and restrain macrophage inflammatory differentiation through the regulation of factors that control macrophage tolerance and inflammation.

Our result shows that anti-VISTA treatment could significantly augment the magnitude of LPS tolerance *in vivo* and *in vitro*. *In vivo*, concurrent anti-VISTA agonist treatment with low-dose suppressed LPS-induced lethality. Despite this, anti-VISTA pretreatment alone did not fully substitute for low dose LPS in inducing LPS tolerance, similar to other anti-inflammatory molecules like IL-10 (78). In contrast, we observed a tolerogenic impact of anti-VISTA agonist pretreatment *in vitro* on reducing subsequent responses to LPS indicating that the tolerogenic effect of VISTA monotherapy indeed is evident, but does not override high-dose LPS *in vivo*. These tolerogenic findings of anti-VISTA pretreatment were seen using both human and murine macrophages suggesting a conserved cross-species role for VISTA. The *in vivo* conditions also speak to the involvement of multiple myeloid populations in promoting the LPS lethal inflammation whereas the *in vitro* systems allow for specific reprogramming of macrophages.

We also present the finding that VISTA agonist induced the development of a regulatory phenotype from resting macrophage independent from and prior to inflammatory stimulation (Figures 1, 3). This result speaks to the constitutive function of VISTA in maintaining immunologic quiescence in the macrophage lineage. High-resolution time-course RNA-seq analysis coupled with proteomic analysis revealed a regulatory profile that was induced by anti-VISTA. Anti-VISTA induced a tolerogenic and anti-inflammatory functional and transcriptional profile in both mouse and human macrophages. The resulting profile of anti-VISTA alone (rapid decrease IRF5 and IRF8 and increased MerTK proteins) was associated with transcriptional upregulation of mediators of tolerance (IRG1, A20). IRF5 has a critical role in macrophage inflammatory polarization, as it influences macrophage activation toward an inflammatory trajectory by direct upregulation of IL-12 and repression of IL-10 genes (9, 60). IRF8 plays similar roles in pro-inflammatory programming of macrophage polarization (61, 62). More recent work showed that IRF5 interaction with NFkB plays a substantial role in the induction of inflammatory genes upon LPS stimulation (63). Reduction in NFkB transcriptional activity in anti-VISTA-treated macrophages suggests that VISTA may be operating upstream of these mediators. By coordinately downregulating these three factors, VISTA signaling restrains macrophages from an M1-like inflammatory response and increases resistance to endotoxin shock.

When concurrently administered with an inflammatory signal, anti-VISTA altered the trajectory of the macrophage inflammatory response to LPS in both magnitude and quality. In the presence of LPS stimulation, anti-VISTA triggered macrophages maintained a profile similar to reprogramming by immunomodulatory stimuli such as glucocorticoids, immune complexes and PGE2 (14). Indeed, this comparison showed that the profile of macrophages after VISTA activation followed by LPS stimulation clustered closer to unstimulated macrophages compared to tolerized or untolerized macrophages, underscoring the profound checkpoint regulation imposed by anti-VISTA on the development of inflammation. Similar results were seen with human monocyte-derived macrophages, suggesting that VISTA represents an evolutionarily conserved negative regulator of macrophage inflammatory responses that exerts a more global impact than that which is seen in LPS tolerance. Strikingly, anti-VISTA reprogramming was also resistant to inflammation driven by IFNγ, consistent with the observation that anti-VISTA resulted in more global reprogramming than seen with LPS tolerance alone. Likewise, anti-VISTA reprogramming resulted in impaired commitment of macrophages toward an M1 pro-inflammatory phenotype thus placing VISTA at the center of negative regulation of macrophage responses. Together, our findings show that VISTA is an important checkpoint for macrophage inflammatory response and agonistic anti-VISTA antibodies could represent an unprecedented asset for modulating myeloid mediated inflammation in human immune-driven diseases.

## Data Availability Statement

RNA-seq and scATAC-seq data are available at accession number GSE157518.

## Ethics Statement

The studies involving human participants were reviewed and approved by Institutional Review Board of Dartmouth College. The patients/participants provided their written informed consent to participate in this study. The animal studies was reviewed and approved by Institutional Animal Care and Use Committee of Dartmouth College, NH, USA (protocols 2012 and 2014). Human monocyte experiments were performed using peripheral blood from healthy male and female volunteers (25 to 40 years). The protocol was approved by the Institutional Review Board of Dartmouth College and conducted in accordance with the ethical principles of the Declaration of Helsinki and Good Clinical Practice as defined by the International Conference on Harmonization. All donors gave written informed consent.

## Author Contributions

RN, ME, and RM: conceptualization. RN, ME, ES, and NS: methodology. ME, ES, RM, and NS: investigation. RN, ME, RM, ES, BB, CB, and CC: writing–review and editing. RN, RM, and CC: resources. RN: supervision. All authors: contributed to the article and approved the submitted version.

## Conflict of Interest

RN is an inventor on patent applications (10035857, 9631018, 9217035, 8501915, 8465740, 8236304, and 8231872) submitted by Dartmouth college, and patent applications (9890215 and 9381244) submitted by Kings College London and Dartmouth College and a cofounder of ImmuNext, a company involved in the development of VISTA-related assets. These applications cover the use of VISTA targeting for modulation of the immune response. The remaining authors declare that the research was conducted in the absence of any commercial or financial relationships that could be construed as a potential conflict of interest.
