# Supplementary figures and images for: VISTA Re-programs Macrophage Biology Through the Combined Regulation of Tolerance and Anti-inflammatory Pathways
Source: Front Immunol. 2020 Oct 15;11:580187. doi: 10.3389/fimmu.2020.580187 (PMC7593571; doi:10.3389/fimmu.2020.580187)

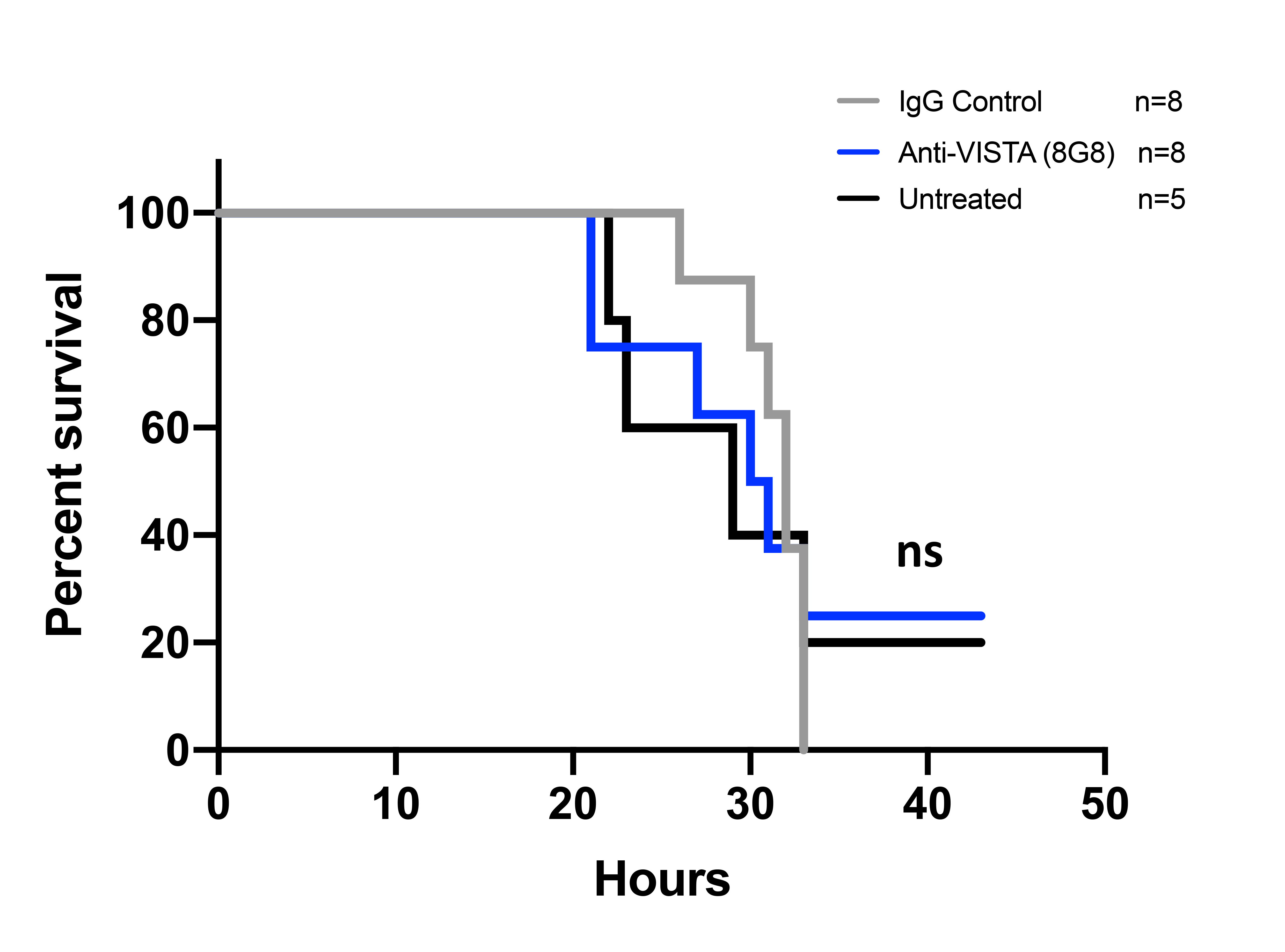

Supplement: Supplementary file 1 [file Image_1.JPEG]

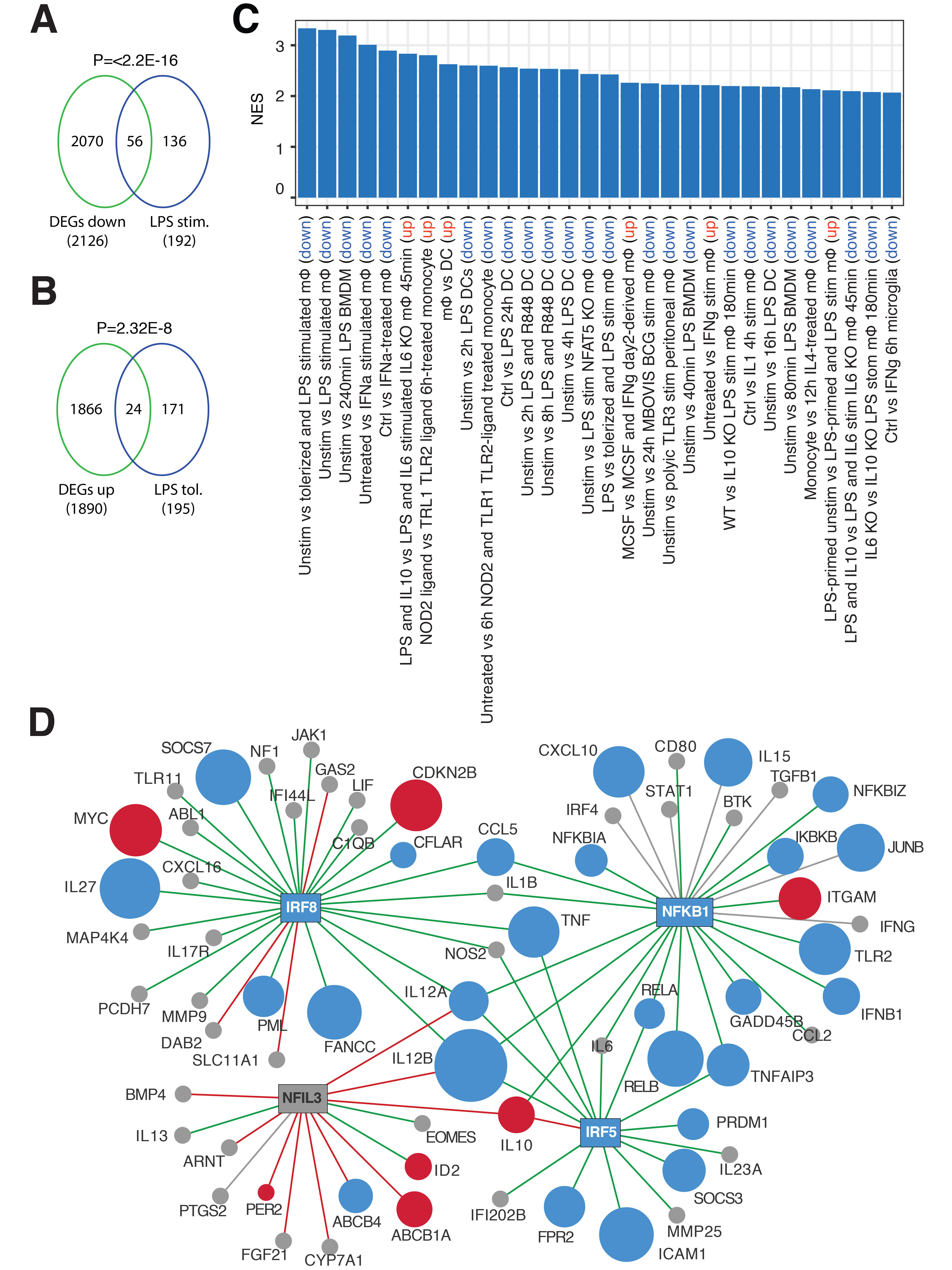

Supplement: Supplementary file 2 [file Image_2.JPEG]

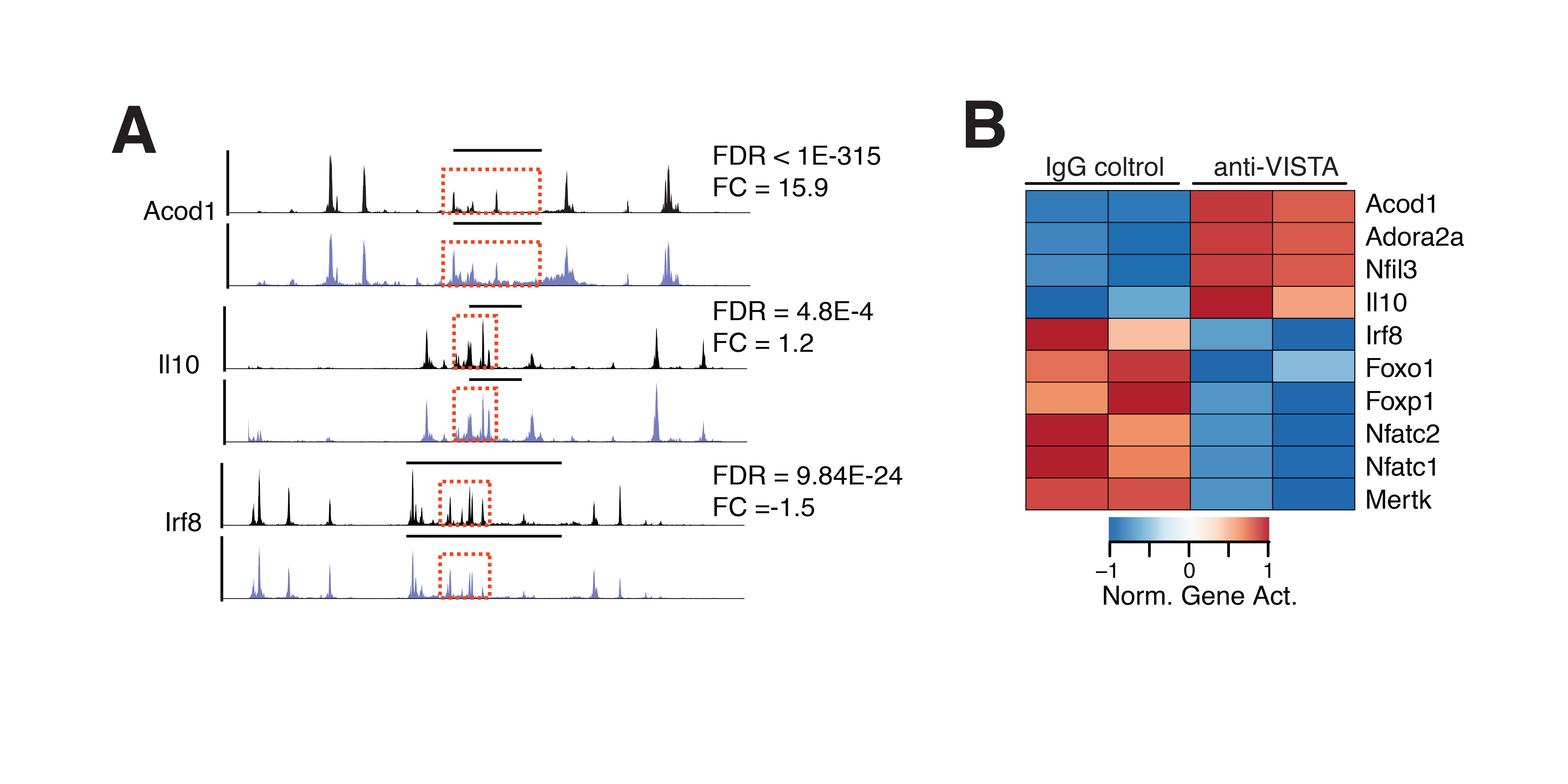

Supplement: Supplementary file 3 [file Image_3.JPEG]

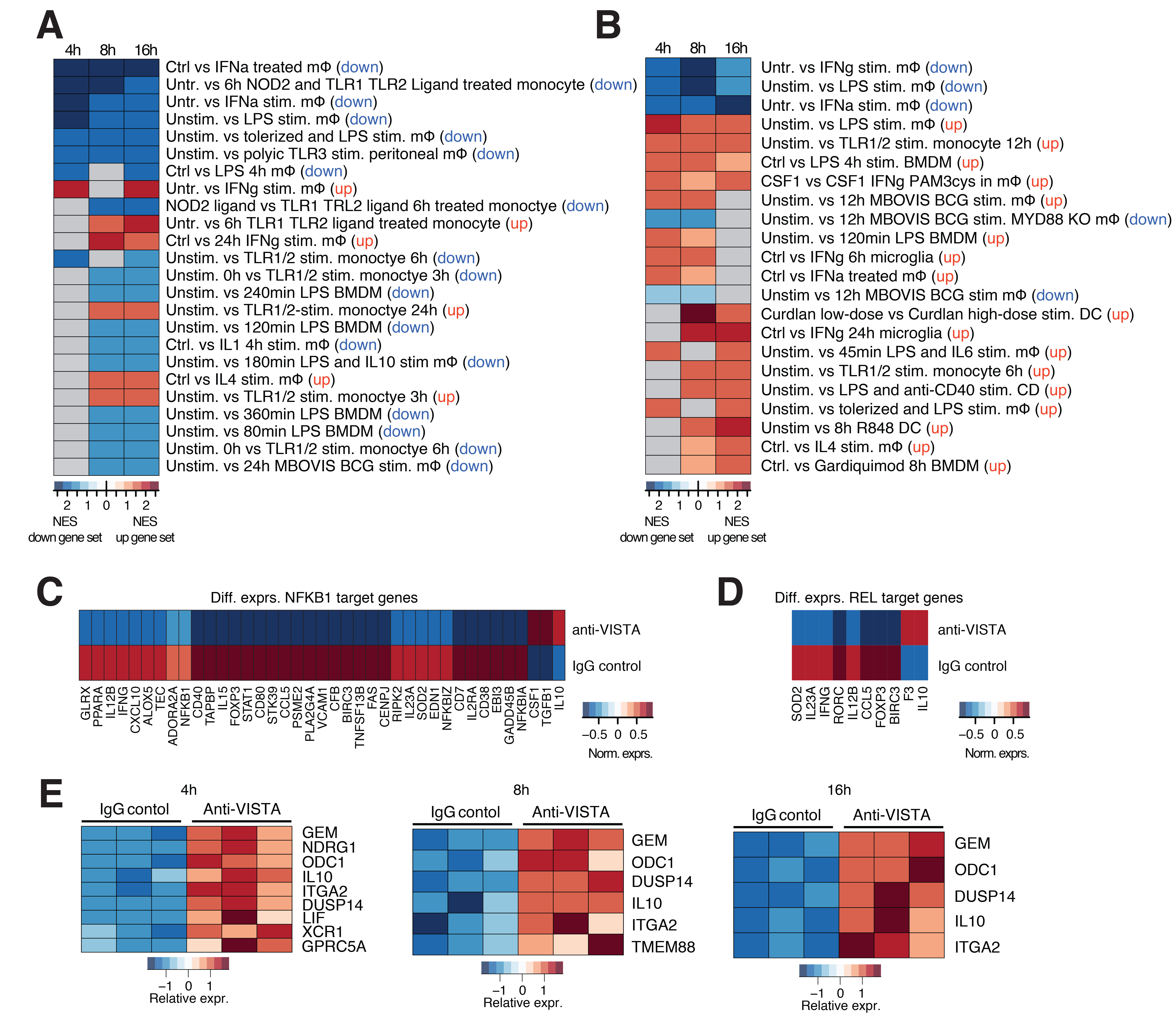

Supplement: Supplementary file 4 [file Image_4.JPEG]
